# An interpretable statistical approach to photovoltaic power forecasting using factor analysis and ridge regression

**DOI:** 10.1038/s41598-025-22838-x

**Published:** 2025-11-06

**Authors:** Vedat Esen, Berhan Coban, Bahar Yalcin Kavus, Tolga Kudret Karaca, Taner Dindar, Ali Samet Sarkin

**Affiliations:** 1 Department of Electric Electronics Engineering, Istanbul Topkapi University, 34087 İstanbul, Turkey; 2 Statistical Consultancy Assessment and Evaluation Research and Application Center, İzmir Katip Çelebi University, 35620 İzmir, Turkey; 3 Quality Coordination Office, İzmir Katip Çelebi University, 35620 İzmir, Turkey; 4 Department of Industrial Engineering, Istanbul Topkapi University, 34087 İstanbul, Turkey; 5 Department of Electronic and Automation, Ankara University, Ankara, Turkey; 6 Department of Electrical and Energy, Osmaniye Korkut Ata University, 80750 Osmaniye, Turkey

**Keywords:** Photovoltaic power generation, Solar power forecasting, Hierarchical time series factor analysis, Ridge regression, Dimension reduction, Energy science and technology, Engineering, Mathematics and computing

## Abstract

Accurate forecasting of solar energy is essential for balancing supply and demand, enhancing energy planning, and supporting the integration of renewable resources into modern electricity grids. While recent research has heavily focused on machine learning-based models such as Long Short-Term Memory networks for solar energy forecasting, these approaches often lack transparency and interpretability. This study presents an interpretable by design photovoltaic (PV) forecasting framework that couples hierarchical factor analysis (HFA) with ridge regression. HFA compresses high dimensional meteorology into three physics meaningful second order factors after which a single parameter ridge model provides coefficient level transparency and regularization in this compact space. Using 15 min measurements from a 93.6 kWp plant in Adıyaman, Türkiye (May 17, 2021–Jan 12, 2025), we evaluate under a unified chronological split (0.64/0.16/0.20). The model combines strong generalization with clear insights into how meteorological variables affect solar power generation, ensuring transparency and verifiability. These results highlight regression-based methods as robust, explainable alternatives to complex deep learning models in photovoltaic forecasting.Since development and forecasting using highly multivariate models is typically not an easy task, our approach is designed to provide a more streamlined model through which future prediction is easier. Simplifying complexity and making it easier to understand how parameters affect the result, our proposed model simplifies finding the most important drivers of solar power generation.

## Introduction

 Renewable energy (RE) is recognized as the key component of the future energy landscape due to the accelerating impact of fossil fuels on climate change^1^. Solar energy, as a sustainable and fast-growing RE source, converts sunlight directly into electricity via PV devices^2^. PV systems are favored for power generation because of their advantages like storage capacity, environmental friendliness, simple design, and grid integration^3^. As PV capacity grows, managing generated power and ensuring system reliability become increasingly critical^4^. While PV module characteristics can be tested in labs under standard conditions (American Society for Testing and Materials (ASTM) 927 − 10), real-world performance is affected by sunlight intensity and atmospheric factors such as temperature, humidity, and wind^5,6^. The variability of meteorological conditions poses challenges for stable and reliable PV system operation and grid integration^7^. Accurate PV power forecasting helps balance electricity supply and demand, reduces grid fluctuations, avoids unnecessary costs, and supports efficient energy storage and grid management, particularly for large-scale solar plants^8–11^.

In this study, a novel two-stage methodological framework is proposed to enhance PV power forecasting by combining HFA and Ridge Regression, with a specific focus on model interpretability, statistical validity, and practical applicability. Unlike the majority of recent studies that predominantly rely on machine learning (ML) and deep learning (DL) techniques, often perceived as black-box models, this study demonstrates that regression-based forecasting can offer not only competitive performance but also superior explainability and verifiability. The HFA is applied in the first stage of the framework-the process targets analyzing and extracting latent factors from enormous meteorologic variables to reduce their complexity while retaining the structural and temporal relationships essential to those variables. These latent factors are then used as inputs to a Ridge Regression model in the second stage, which mitigates multicollinearity and improves generalization through L2 regularization.

This approach challenges the current trend in the PV forecasting literature by showing that interpretable regression models, when combined with robust dimensionality reduction techniques, can outperform or rival more complex ML algorithms, particularly in terms of model transparency, traceability, and reproducibility. This study makes three main contributions to the field of PV power forecasting:

1. It introduces a hierarchical structure that reduces dimensionality and preserves temporal and thematic relationships among features.
2. It applies an interpretable and regularized linear regression method, which allows for transparent prediction modeling and stability under high correlation.
3. Based on actual PV power data from a solar power plant in Türkiye, the applicability and effectiveness of the proposed framework are demonstrated, which in turn establishes its significance in terms of generalizability, integration into the grid, and energy planning.

## Literature review

In PV power forecasting, three main estimation techniques are widely used. The first is physical approaches that model energy production based on meteorological variables and photovoltaic device interactions, often employing numerical weather prediction, sky imaging, and satellite data^12^. The second involves statistical and ML methods, which are fast, efficient, and cost-effective for short- and medium-term forecasts^13^. These include time series analysis, ML, and DL techniques^14^. Time series models, such as ARIMA, have proven effective for short-term forecasts in large-scale PV plants^15^. ML models like Artificial Neural Networks (ANN), Random Forests, Support Vector Machines (SVM), Gaussian Process Regression (GPR), and Extreme Learning Machines (ELM) are popular due to their ability to handle nonlinearity and heterogeneous inputs^8,16^. Ensemble and optimized ML models have shown improved accuracy; for instance, ANN ensembles, Random Forest after feature selection, and GPR were found most accurate in various studies^17–19^. Optimized ELM models have also demonstrated stable short-term forecasts in microgrids^20^. Due to scalability challenges with large PV datasets, DL models like Recurrent Neural Networks (RNN), Long Short-Term Memory (LSTM), Gated Recurrent Units (GRU), and hybrid CNN–LSTM architectures are increasingly used^12^. While early studies used standalone RNNs^21^, hybrid models currently dominate for better performance^22^. Examples include RNN-LSTM combined with physical models achieving high accuracy in France^23^, a 4-layer LSTM model with low error in Brazil^24^, and a CNN-GRU hybrid for a floating PV plant in Thailand^25^. Hybrid approaches combining physical and statistical methods continue to gain attention, often involving data preprocessing, model building, and parameter tuning^26^. Studies report hybrid models outperform standalone ones in one-week forecasts^27^. Recent reviews emphasize managing uncertainty, improving data quality, and enhancing generalizability in ML-based renewable forecasting^28^. Advanced hybrid frameworks incorporating feature selection, signal decomposition, and DL optimization, have achieved high accuracy on large PV systems^26,29^. Besides nonlinear ML and DL models, traditional regression methods are also explored. For example, comparisons of multiple regression and ML models on Jordanian PV data with Chimpanzee Optimization Algorithm tuning showed MLP provided the best forecasting accuracy^16^.

Recent studies on PV power forecasting highlight the importance of both methodological innovation and explainability. Research on building-integrated PV systems in Switzerland has shown that careful model tuning and evaluation can significantly improve predictive performance. A weighted ensemble combining machine learning models with sky imagery demonstrated superior short-term forecasting accuracy compared to traditional approaches^29^. Nematzadeh and Esen proposed an explainable ML framework that identifies the most influential meteorological parameters and delivers generalizable forecasts without relying on site-specific sensors^30^. Tripathi et al. compared multiple regression techniques under fluctuating weather conditions and found Support Vector Machine Regression (SVMR) to outperform both multivariate and Gaussian regression^31^. comprehensive IEEE Access review emphasized the growing role of ensemble methods such as Random Forest and Boosting for short-term PV forecasting^32^. Complementing this, studies dedicated to SVMR confirmed its ability to capture nonlinear dependencies and provide highly accurate PV power predictions^31^.

Beyond methodological advances, PV forecasting research also extends to diverse application contexts such as BIPV and FSPV systems, where unique environmental challenges further highlight the need for robust and interpretable models. PV power forecasting studies can be conducted not only for power plants but also for BIPV (Building Integrated Photovoltaic) systems and FSPV (Floating Solar Photovoltaic) systems, which can be installed as alternatives in densely populated locations where land is scarce. BIPV systems are created by installing solar PV-based power systems on traditional structures^33,34^. FSPV plants are systems installed on water surfaces and aim to increase production performance by eliminating land acquisition^35^. Partial shading problems, physical properties of the environment, and many problems arising from objects can complicate forecasting studies in BIPV systems^36^. In FSPV systems, unlike land, this difficulty is caused by factors such as water temperature, ambient humidity, panel temperature, and the cooling effect. Consequently, the sensor data used in these systems often contains multicollinearity. Therefore, Factor Analysis is used to identify critical parameters, and Ridge Regression is used to address the multiple correlation problem in BIPV and FSPV. The methods, strengths, and limitations of key studies in the literature together with application contexts such as BIPV and FSPV are summarized in Table 1.

**Table 1:** Comparative summary of existing research on PV power forecasting.

Category/Study	Typical methods	Merits	Limitations	Ref.
Physical/ mechanistic approaches	NWP-driven irradiance PV conversion	Physics-grounded; can work with limited historical time-series; transparent assumptions	Sensitive to NWP errors; needs detailed inputs/sensors; calibration overhead	^29,^ ^35^
Time-series (AR/ARIMA/ARIMAX)	Univariate/multivariate ARIMA baselines	Simple, fast, strong at short horizons; useful benchmarks	“Inertia” under rapid variability; accuracy drops as horizon grows/sky changes	^13-15^
Classical ML (ANN, RF, SVM, GPR, ELM)	Nonlinear learners on engineered features	Handle heterogeneity & nonlinearity; often beat statistical baselines	Tuning/feature engineering; overfitting risk; mixed interpretability	^12,21,22,38^
Ensembles	ML + sky-imager + ARIMA blended; ridge-stacked weights	Consistently higher skill than constituents; robust in variable skies	Multi-stream data (imagery + radiometry) and higher implementation complexity	^8,16^
Deep learning (RNN/LSTM/GRU/LSTM)	Sequence models; image-aware CNN hybrids	Strong accuracy with large data; captures temporal–spatial patterns	Data/compute hungry; black-box behavior limits explainability	^23-25,28^
Hybrid physical + data-driven	Two-step irradiance forecast + empirical PV model; ML with exogenous physics	Combines complementary strengths; improves generalization	Pipeline complexity; error propagation must be handled carefully	^26,27^
Interpretable/ XAI frameworks	Feature selection + SHAP; factor selection + transparent regressors	Identifies influential drivers; auditability; deployment-friendly	Sometimes slightly below best deep ensembles in raw accuracy	^36, 37^
BIPV application studies	Model tuning, grid search, K-fold on BIPV strings	Shows performance gains from rigorous tuning/evaluation	Site-/plant-specific constraints; reliance on external met inputs	^33-36^
SVMR vs. classical regression	Direct comparison: SVMR, Gaussian regression, multivariate regression	SVMR best captured nonlinearities; lowest MAE/MSE; high R2R^2	Kernel/tuning sensitivity; less transparent than linear models	^31,32,39^
Reviews/syntheses	Taxonomy & benchmarks (ML, ensembles, DL)	Situates best-in-class methods; highlights ensembles’ short-term gains	Heterogeneous datasets hinder strict apples-to-apples comparisons	^35^
Interpretable PV forecasting with (HFA + Ridge)	HFA to reduce meteorological and Ridge Regression	Interpretability, Robust to multicollinearity, Overfitting resistance, Low cost/ easy deployment	Factor stability assumptions, No probabilistic output, Nowcasting under fast cloud dynamics, Input quality sensitivity	Proposed Method

Although time series forecasting and machine learning-based approaches have been extensively explored in the context of PV power prediction, the integration of dimensionality reduction techniques within time series frameworks remains significantly underrepresented in the literature. Moreover, studies that apply factor reduction are predominantly aligned with black-box ML models, while the potential of interpretable and statistically rigorous models has not been adequately examined. In light of this gap, the present study proposes a novel two-stage framework: initially employing Hierarchical Time Series Analysis to model temporal dependencies while simultaneously reducing feature dimensionality, followed by the application of Ridge Regression to the refined factor set for accurate, generalizable, and interpretable PV power forecasting.

## Methodology

**Fig. 1:**
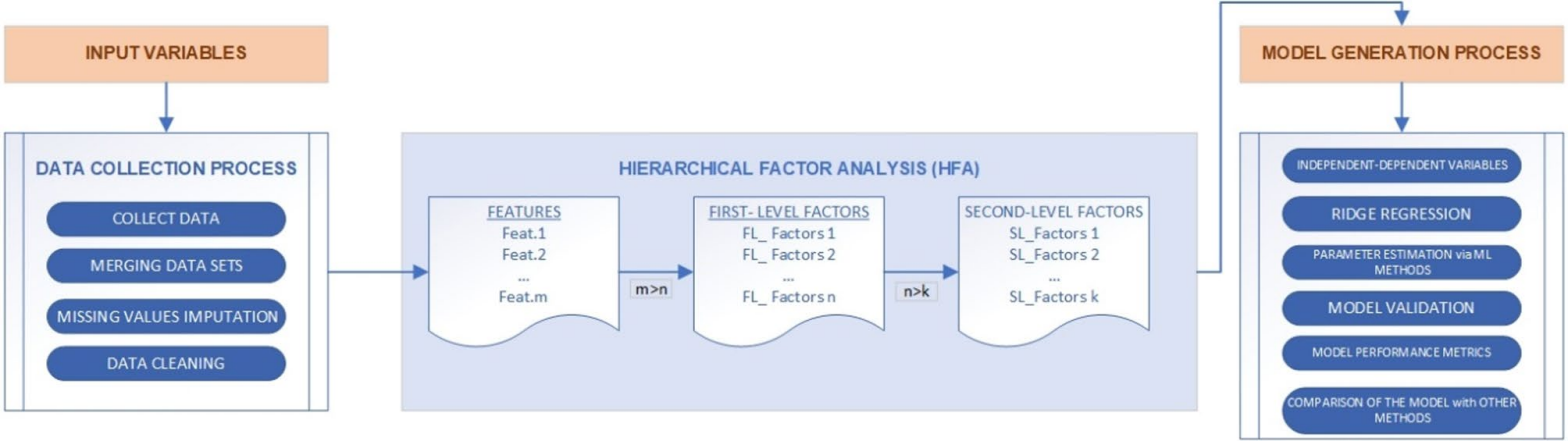
Structure of the proposed methods.

This study employed HFA and Ridge Regression methods to address the high dimensionality of time series data and improve the modeling process (Fig. 1). Initially, the Time Series Factor Analysis (TSFA) technique was used to transform the high-dimensional data into a more manageable structure by identifying latent constructs among all observed variables. The components obtained from the factor analysis were subsequently included in the model using the Ridge Regression method to evaluate their effects on the dependent variable. This section provides an overview of the dataset, including data source and factors characteristics, and brief descriptions of the analytical methods used.

### Hierarchical time series factor analysis (HFA)

HFA is a multi-stage statistical procedure developed to uncover latent structures within complex and high-dimensional datasets, such as time series, and to facilitate a deeper interpretation of inter-variable relationships. HFA, unlike traditional ones, is not only to find the direct relationships between observed variables and latent factors but also at relationships that directly between extracted factors. The hierarchical structure thus proves to be of great importance in terms of theoretical clarity and statistical robustness. In the initial stage of HFA, factor loadings are estimated using techniques such as Principal Component Analysis (PCA) or the Maximum Likelihood method. These loadings extract a set of first-order latent factors that capture the primary shared variance among the observed variables. The next step, and the second stage, establishes the intercorrelations among the first-order factors to identify higher-order latent constructs. Hence comes about a multi-level structure of factors that parsimoniously summarizes the underlying data^37^.

With this staged dimensionality reduction approach, both the understandability of the model increases and a critical problem like multicollinearity, which usually encounters regression analyses with highly correlated predictors, is satisfactorily addressed. Latent constructs will help reduce the number of variables while retaining a significant variance, as HFA makes the ensuing modeling phases statistically more efficient and informative^38^.

The methodological application of HFA follows a systematic sequence of steps. The procedure starts with data preparation, where the dataset gets preprocessed through normalization and treatment of missing values to meet the assumptions required for factor analysis. Following this, exploratory factor analysis is applied to the observed variables to extract a reduced number of first-order latent constructs. Determining the optimal number of these factors involves using statistical heuristics such as the Kaiser criterion (eigenvalue > 1), scree plot examination, and parallel analysis. To improve the clarity and interpretability of factor structures, rotation techniques are employed- either orthogonal methods like Varimax or oblique alternatives such as Promax- depending on the nature of factor correlations. Once the first-order factors are established and refined, a second-level factor analysis is conducted on their intercorrelation matrix to identify broader, second-order latent dimensions. Finally, the hierarchical model is evaluated using various goodness-of-fit indices, which collectively assess the adequacy and validity of the proposed structural hierarchy^39^.

### Ridge regression

Ridge Regression was introduced by Hoerl and Kennard to address the problem of multicollinearity in the classical multiple linear regression model. Especially when there is a high correlation between independent variables, the regression coefficients estimated by the classical Ordinary Least Squares (OLS) method may show high variance and this may negatively affect the generalization capacity of the model. Ridge Regression aims to solve this problem by adding an L2 penalty term. Ridge Regression mitigates this issue by applying a shrinkage approach, introducing a small amount of bias to reduce variance. ^40^. The classical linear regression model is defined as:

1 $$\:y=\beta\:X+\epsilon\:$$

where y is a vector of the dependent variable, X is a matrix of independent variables, β is a vector of regressions coefficients and ε is the error term. The OLS estimator is given by:

2 $$\:{\widehat{\beta\:}}_{OLS}={\left({X}^{T}X\right)}^{-1}{X}^{T}y$$

However, when the matrix X^T^X is nearly singular, the variance of this estimator increases. To counter this, Ridge Regression introduces a penalty term to the objective function:

3 $$\:{\widehat{\beta\:}}_{ridge}=\underset{\beta\:}{\text{argmin}}\left\{\parallel{y-\beta\:X\parallel}^{2}+k{\parallel\beta\:\parallel}^{2}\right\}$$

Here, k ≥ 0 is referred to as the Ridge parameter and controls the degree of regularization. The Ridge solution is obtained as:

4 $$\:{\widehat{\beta\:}}_{ridge}={\left({X}^{T}X+k\text{I}\right)}^{-1}{X}^{T}y$$

This formulation penalizes the sum of squared coefficients, discouraging large weights and resulting in a more stable and generalizable model. Ridge Regression can outperform OLS in terms of mean squared error (MSE), particularly when multicollinearity is present.

## Experimental results

This section presents the methods of data collection for the implementation of the proposed methodology and the findings obtained as a result of the methodology.

### Data collection

**Fig. 2:**
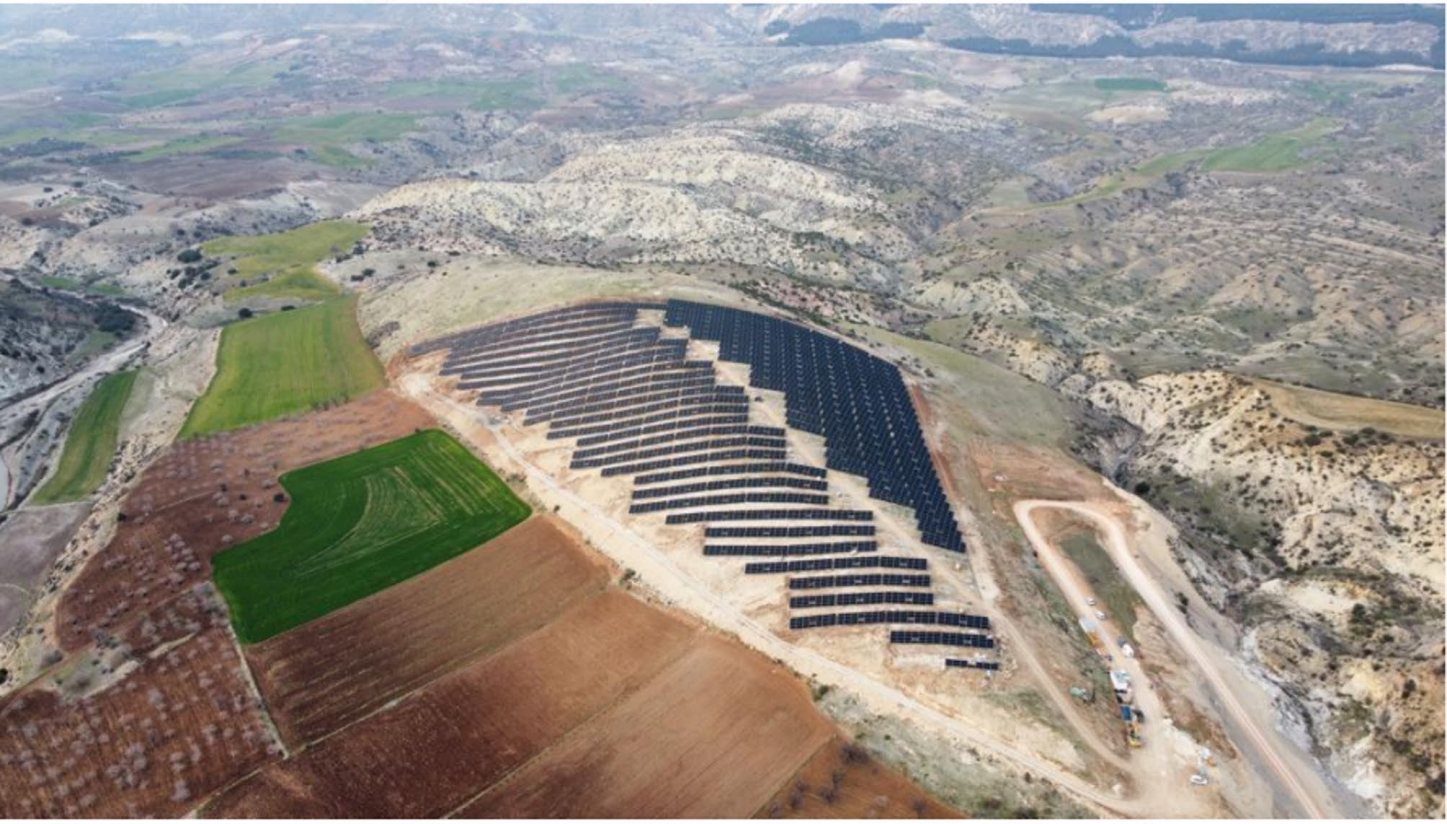
The power plant image employed in the study.

The dataset consists of time series data collected from a PV power plant with 93,6 kWp (kilowatt-peek) installed in Besni, Adıyaman, Türkiye. Besni is located in southeastern Türkiye, on the western side of Adıyaman Province. Adıyaman is located at 38° 18’ 11.40” east longitude and 37° 48’ 1.19” north latitude. Many PV power generation systems have been installed in Adıyaman. In this study, among the PV systems, the system located in Besni, located between 38° 18’ 48” east and 37° 43’ 15” north, was selected as the system that best meets the ideal standards for data collection (Fig. 2). All data used for training and evaluating the models in this study are freely available in a public GitHub repository. It is accessible at: https://github.com/tkaraca/An-Interpretable-Statistical-Approach-to-Photovoltaic-Power-Forecastin.git.

A total of 234 400 Wp monocrystalline PV panels were used in the system. The electrical properties of the panels used are given in Table 2.

**Table 2:** PV panel electrical characteristics.

Peak Power (Pmax)	400 Wp
Module Efficiency (%)	20,06
Maximum Power Voltage (Vmp)	41,10
Maximum Power Current (Imp)	9,75
Open Circuit Voltage (Voc)	49,83
Short Circuit Current (Isc)	10,38
Power Tolerance	0 ~ + 5 W
Operating Temperature	−40 ~ + 85 °C

In the database provided by these PV systems, data that has not been used in any previous study was recorded from the grid-connected power plant between 17/05/2021 and 12/01/2025. Data obtained from the facility includes active power data of photovoltaic panels. All data were recorded every 15 min. Meteorological data were taken from the Open Meteo website on dates and time intervals synchronous with the PV data.

Based on the data obtained, a total of 20 features are identified. Active Power represents the product of the voltage and current generated by solar radiation on the PV cell along with the power factor, indicating the efficiency of power usage. Total Irradiance is the amount of electromagnetic energy from the Sun reaching the Earth’s atmosphere and directly influences PV power generation as it increases. Temperature rise reduces the open-circuit voltage of the PV panel while causing a slight increase in short-circuit current. Relative Humidity negatively impacts energy received due to dew droplets reflecting solar radiation in different directions. The Dew Point is the temperature at which humid air becomes saturated and forms dew or fog. Wind Speed positively affects PV performance by cooling the modules, whereas the effect of Wind Direction varies depending on the hemisphere. Wind Gusts refer to sudden fluctuations in atmospheric wind and are considered along with speed and direction. Visibility, reduced by gases, water vapor, and dust, decreases solar radiation reaching the panels and thus lowers energy production. Precipitation increases surface wetness, impacting PV output. Apparent Temperature describes the temperature perceived by the human body. Shortwave Radiation covers solar radiation in the 300–3000 nm wavelength range. Direct Radiation is sunlight reaching the earth without scattering, while Diffuse Radiation consists of scattered or absorbed solar radiation due to atmospheric particles. Direct Normal Irradiance illuminates a surface perpendicular to the sun’s rays. Global Tilted Irradiance quantifies the total solar energy received by a sloped surface, useful for evaluating fixed-tilt PV panels. Terrestrial Radiation is the long-wave, low-energy radiation emitted by the Earth. Snowfall Height indicates snow accumulation that negatively affects PV system performance in snowy regions. Freezing Level Height marks the altitude where air temperature is 0 °C. Lastly, Sunshine Duration measures the total time the earth is exposed to direct solar radiation.

### Application of HFA

The original dataset, composed of 29 meteorological and environmental factor**s**, is subjected to HFA to reduce its dimensionality in a structured and interpretable manner. In the first stage, the data are compressed into nine first-level factors, which are subsequently grouped into three second-level factors, forming a hierarchical structure. Before factor reduction, correlation matrix and logical associations among variables are examined for factor analysis suitability. The number of factors to retain is finally determined by using different statistical heuristics: the eigenvalue criteria (λ > 1) and a scree plot.

**Fig. 3:**
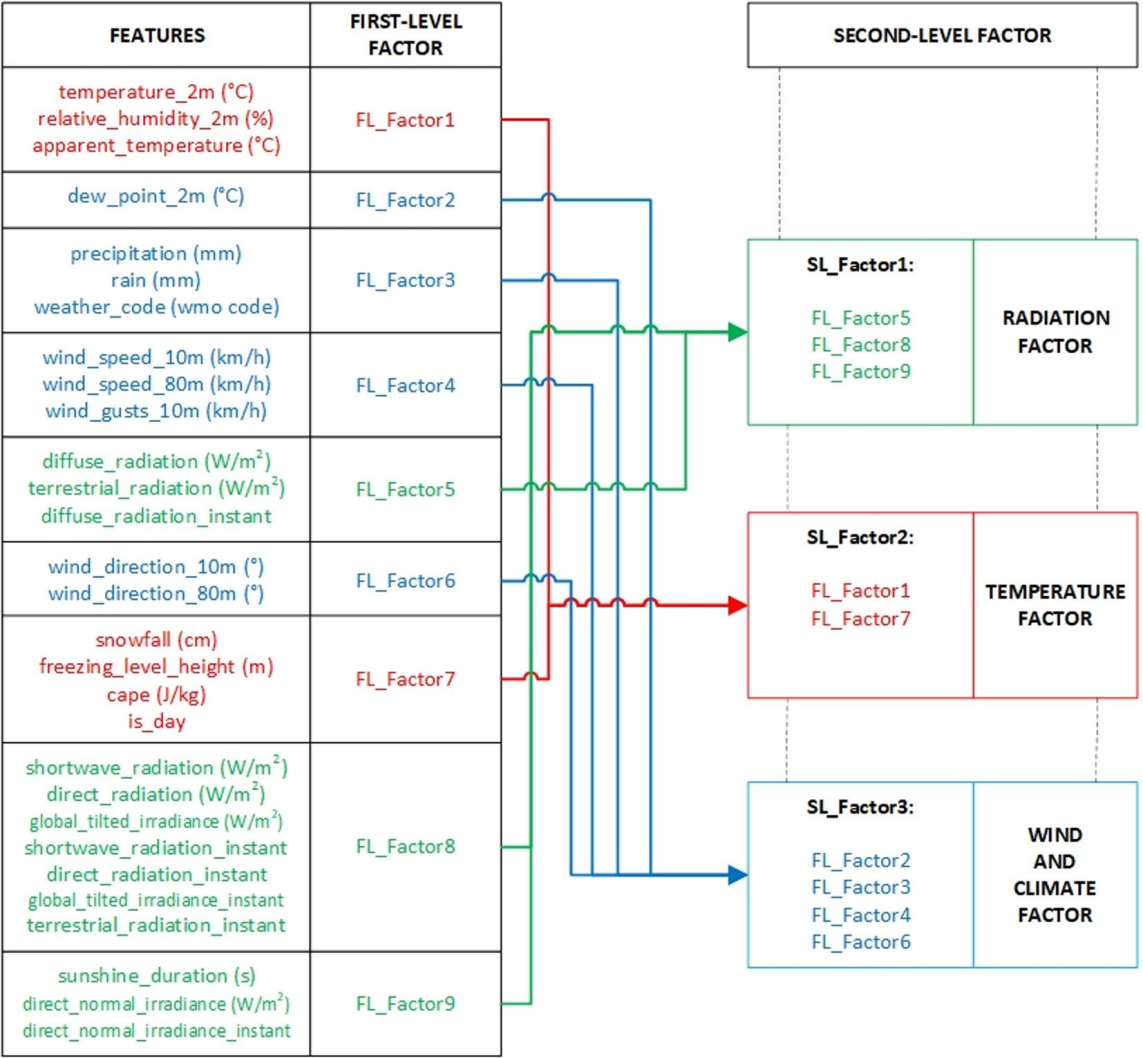
Two-Level factor analysis.

The resulting first level factors (FL_Factor1–FL_Factor9) grouped thematically and statistically similar indicators. In the second stage, the correlations among these factors are analyzed to derive broader, conceptually cohesive second-level factors, as illustrated in Fig. 3.

The second-level factor structure derived from the first-level factors was designed to capture broader meteorological themes by aggregating conceptually and statistically related components.

- SL_Factor 1, labeled the “Radiation Factor”, encapsulates all irradiance-related variables and includes FL_Factor5, FL_Factor8, and FL_Factor9.

- SL_Factor 2, called the “Temperature Factor”, comprises thermal and humidity-related indicators and FL_Factor1, FL_Factor2, and FL_Factor7.

- SL_Factor 3, termed the “Wind and Climate Factor”, integrates wind characteristics, precipitation data, and other general atmospheric parameters through FL_Factor3, FL_Factor4, and FL_Factor6.

This hierarchical reduction process preserves the underlying variance present in the big data set and effectively reduces multicollinearity. As a result, it contributes to the development of more stable, generalizable and interpretable regression models in the subsequent estimation phase. To further validate the hierarchical structure, a rotated component matrix is generated to examine the factor loadings of the first-level factors onto second-level factors.

Table 3 summarizes the results of the second-level hierarchical factor analysis by presenting the rotated component matrix, which illustrates how each first-level factor (FL_Factor1 to FL_Factor9) loads onto the second-level latent constructs (SL_Factor1, SL_Factor2, and SL_Factor3). The highest loading values in each row indicate the dominant association of each first-level factor with a particular second-level factor. For example, FL_Factor5, FL_Factor8, and FL_Factor9 load most strongly onto SL_Factor1, corresponding to the radiation factor. Likewise, FL_Factor1, FL_Factor2, and FL_Factor7 are primarily aligned with SL_Factor2, representing the temperature factor. Meanwhile, FL_Factor3, FL_Factor4, and FL_Factor6 are associated with SL_Factor3, forming the wind and climate factor. The rotation technique used was oblimin, which permits correlations between factors and is suitable for meteorological datasets where interdependence among variables is expected. The strength and clarity of the loadings confirm the internal consistency of the factor structure and support the validity of the thematic groupings used in subsequent modeling. This matrix thus provides a statistically reliable and interpretable basis for building regression models that are both accurate and explainable.

**Table 3:** Rotated component matrix of HFA.

First Level Factors	Second Level Factors		
	SL_Factor 1	SL_Factor 2	SL_Factor 3
**FL_Factor 1**	−0,02803	**0**,**861588**	0,2508
**FL_Factor 2**	−0,01933	−0,01305	**0**,**697418**
**FL_Factor 3**	0,048129	0,044879	**0**,**41936**
**FL_Factor 4**	0,055314	0,00147	**0**,**784163**
**FL_Factor 5**	**0**,**656412**	0,009544	0,126775
**FL_Factor 6**	−0,03882	−0,28503	**0**,**882961**
**FL_Factor 7**	−0,05432	**0**,**510029**	0,384738
**FL_Factor 8**	**0**,**925292**	0,032194	−0,00673
**FL_Factor 9**	**0**,**529191**	−0,055	0,020136

### Ridge regression of three factors model

A linear regression model is trained using the L2 (Ridge) regularization method for the obtained three-factor model. Ridge regression penalizes the sum of squared coefficients to control model complexity and prevent overfitting. The selected λ value corresponds to the one that minimizes cross-validated MSE, while maintaining generalizability through the 1-SE rule criterion.

**Fig. 4:**
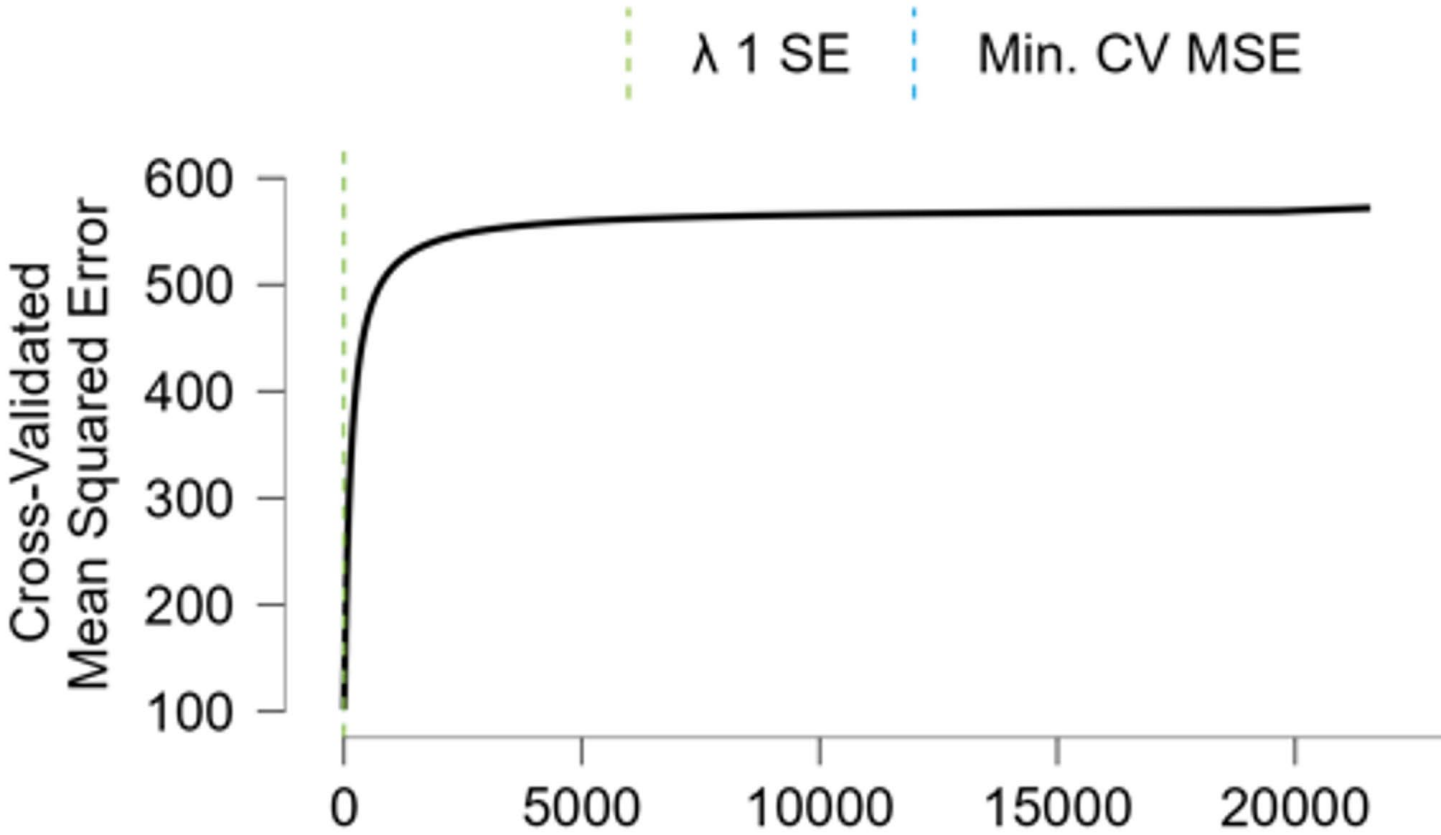
Lambda Selection via Cross-Validated MSE in Ridge Regression.

Figure 4 displays the relationship between different values of the regularization parameter (λ) and the cross-validated MSE in the Ridge Regression model. As λ increases from zero, the MSE initially decreases sharply, indicating improved generalization due to reduced overfitting. However, after a certain point, further increases in λ lead to a plateau and eventually a slight increase in error, reflecting the model’s underfitting tendency.

Two vertical reference lines are included on the plot: the first (blue) corresponds to the λ value that yields the minimum cross-validated MSE, while the second (green) denotes the 1 standard error (1-SE**)** rule, which selects the most regularized model whose error is still within one standard error of the minimum. In this study, the optimal λ value is chosen based on this trade-off to balance complexity and generalizability. The plot confirms that a moderate λ value prevents overfitting without sacrificing much predictive accuracy.

Detailed performance results of the model are provided in Table 2. Ridge regression penalizes the sum of squared coefficients to control model complexity and prevent overfitting. The regularization parameter lambda (λ) is optimized to minimize the MSE on the validation set, and the optimal value is determined as 2.171. The training process includes 45,351 samples for the training set, 11,338 samples for the validation set, and 14,172 samples for the test set. As a result, the MSE values on the validation and test sets are obtained as 104.637 and 104.251, respectively. The small difference between these two error values indicates that the model generalizes well from the validation set to the test set, demonstrating high generalization capability.

The model’s overall performance is further evaluated using various error metrics calculated on the test set (Table 4). In addition to the MSE, the root mean squared error (RMSE) is computed as 10.21, while the mean absolute error (MAE) and mean absolute percentage error (MAPE) are obtained as 7.087 and 8.91%, respectively. Coefficient of determination (R²), the proportion of variance in the dependent variable accounted for by the independent variables of the model, is calculated to be 0.823. It indicates that the Ridge regression model can account for approximately 82.3% of variation in the target variable. Such a high R² value suggests that the model effectively captures the underlying data structure and minimizes unexplained variance. In conjunction with the low error metrics observed (MSE, RMSE, MAE, and MAPE), this value supports the conclusion that the model achieves high predictive accuracy and robust generalizability when applied to unseen data (Table 5).

**Table 4:** Model summary of regularized linear regression.

Penalty		λ		*n*(Train)		*n*(Validation)		*n*(Test)		Validation MSE		Test MSE	
L2 (Ridge)		2.171		45,351		11,338		14,172		104.637		104.251	

**Table 5:** Model performance Metrics.

	MSE	MSE(scaled)	RMSE	MAE / MAD	MAPE	*R*²
HFA–Ridge	104.251	0.186	10.21	7.087	8.91%	0.823

In this additive decomposition, the Base value (29.459) represents the model’s intercept, which remains constant across all cases and reflects the predicted outcome without any feature input. The final Predicted value is computed by summing this base with the weighted contributions of features x_1_, x_2_, and x_3_. The contributions shown in the Table 6 are directly influenced by the coefficients learned by the Ridge Regression model. As Ridge applies L2 regularization, it penalizes large coefficients to reduce overfitting and multicollinearity, distributing the influence more smoothly across correlated features. In this case, x_1_ consistently contributes the most to the prediction, indicating that it holds the highest weight in the model despite regularization.

**Table 6:** Additive explanations for predictions of test set cases.

Case		Predicted		Base		x_1_		x_2_		x_3_	
1		49.990		29.459		20.246		0.180		0.105	
2		60.035		29.459		30.200		−0.056		0.433	
3		61.473		29.459		31.637		−0.094		0.471	
4		63.698		29.459		33.844		−0.105		0.500	
5		65.472		29.459		35.557		−0.026		0.482	

Conversely, x_2_ has negligible contributions, indicating that Ridge has successfully reduced its coefficient towards zero since it is less predictive. x_3_ has positive contributions with moderate effect in all cases, indicating its complementary but secondary nature in the regression equation. These contribution values provide insight into how Ridge Regression allocates influence among features and reinforces the interpretability of linear models when paired with additive explanation methods. Such analysis is valuable for evaluating model performance and understanding which features drive individual predictions.

The regression equation shown in Eq. 1 summarizes the coefficients estimated by the Ridge Regression model. The intercept value of 29.416 is the baseline prediction when all features are zero. Among the features, f_1_ has the highest coefficient (19.274), indicating that it is the most influential predictor in the model. f_2_ has a moderate effect (1.222), while f_3_ has a minimal influence (0.168), which reflects Ridge Regression’s tendency to shrink less relevant coefficients toward zero through L2 regularization.

1 $$\:y=29.416+19.274{f}_{1}+1.222{f}_{2}+0.168{f}_{3}$$

The regression equation summarizes the coefficients estimated by These coefficients directly related to the feature contributions observed in earlier prediction tables, confirming that f1 consistently drives the model’s output. Ridge Regression’s regularization enhances the model’s stability and prevents overfitting, particularly when features are correlated, or the feature space is high-dimensional.

Ridge Regression is selected in this study due to its robustness and suitability for PV power forecasting tasks, where input features such as solar irradiance, temperature, humidity, and time variables are often interrelated and subject to noise. In PV forecasting, model generalizability is critical because environmental conditions vary significantly across time and location. Ridge Regression addresses this by penalizing big values of the coefficients, preventing overfitting of training noise or outliers by the model. It also ensures a balance such that all the predictors contribute proportionally irrespective of multicollinearity, which is common in meteorological data. By stabilizing coefficient estimates and reducing model variance, Ridge Regression enhances prediction accuracy and provides more reliable outputs and is hence a suitable and interpretable model for predicting short-term PV power generation.

**Fig. 5:**
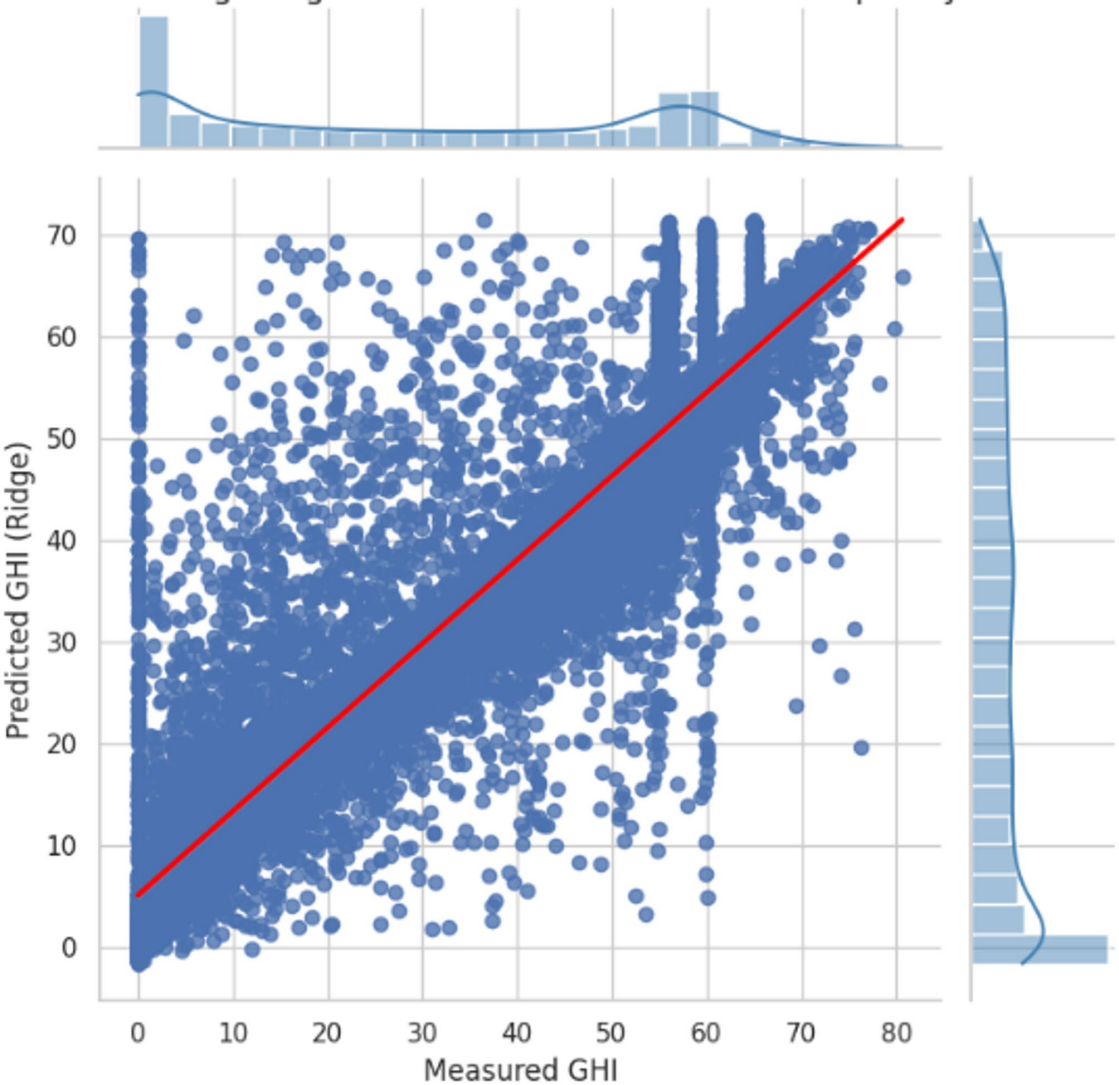
The predictive performance of the Ridge Regression.

Figure 5 illustrates the predictive performance of the Ridge Regression model. The scatter plot compares the predicted values with the measured observations. The red diagonal line represents the ideal case in which predictions perfectly match the measured values. As shown in the figure, the majority of the points cluster around the diagonal, indicating that the Ridge Regression model achieves a strong degree of accuracy.

Despite some dispersion, particularly at lower and higher values, the general alignment suggests that the model captures the underlying trends in the data effectively. The marginal histograms on the axes are also instructive regarding predicted and observed values’ distribution and indicate that the model slightly underpredicts with higher irradiance levels.

**Fig. 6:**
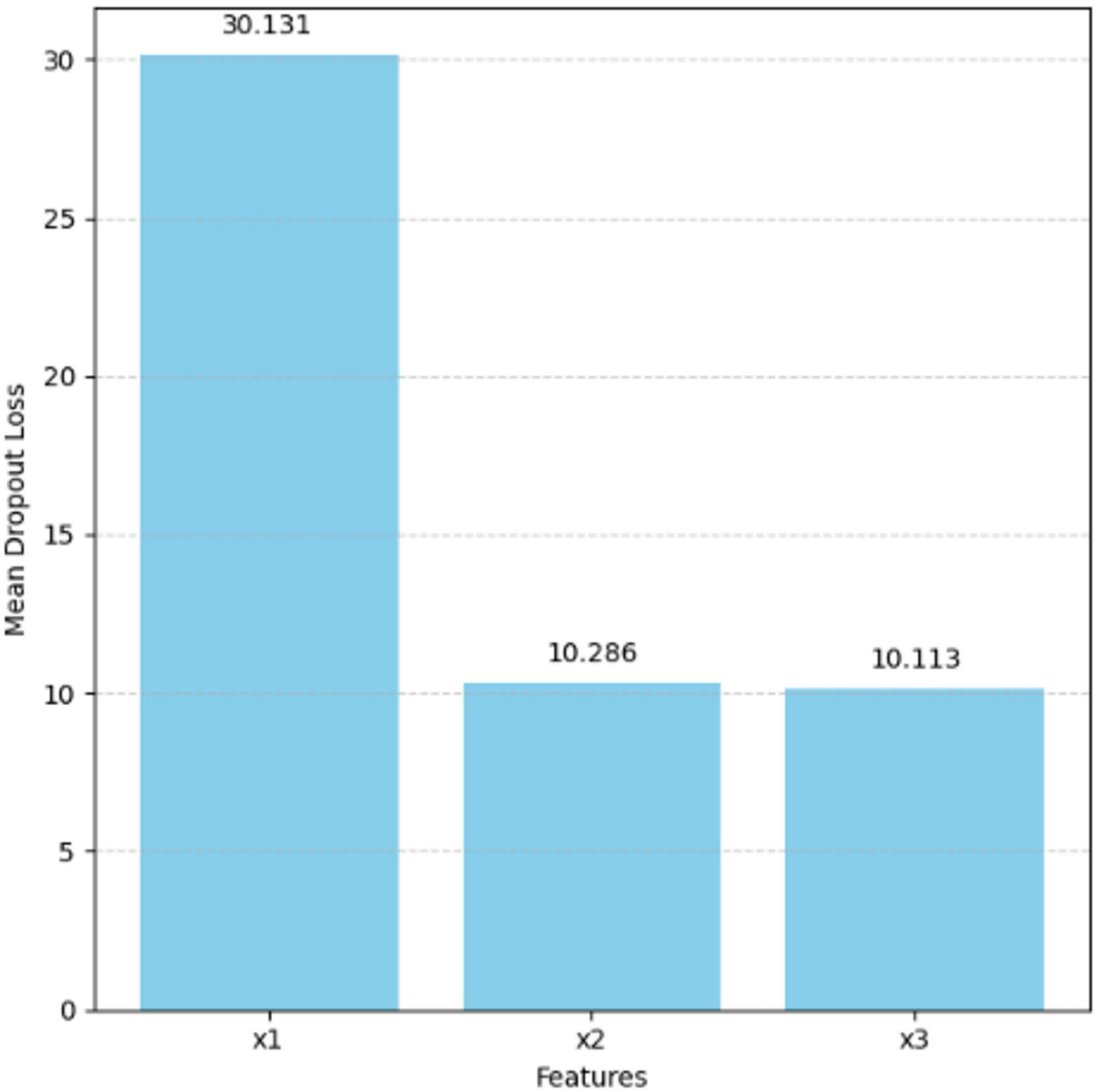
Feature coefficients.

Figure 6 presents the feature importance scores of the Ridge Regression model based on mean dropout loss. Among the features, x_1_ has the largest mean dropout loss (30.13), the most significant feature in making accurate predictions by the model. In the contrary, x_2_ and x_3_ have comparatively lower contributions to the mmodel’sperformance, with dropout losses of 10.286 and 10.113, respectively. In contrast, x_2_ and x_3_ contribute comparably less to the mmodel’sperformance, with dropout losses of 10.286 and 10.113, respectively.

These findings are consistent with the coefficient magnitudes observed in the Ridge Regression equation. The high dropout loss for x_1_ reaffirms its dominant predictive role, while the relatively low values suggest that the model can still perform adequately in their absence. This analysis highlights Ridge Regression’s ability to concentrate predictive weight on the most informative variables while maintaining regularized control over less significant ones, further enhancing generalizability.

## Validation of the study

In this study, a validation process is conducted using real-world data to evaluate the predictive performance of the developed model. In the validation phase, we used a new, independent dataset distinct from the training and testing data. Specifically, the dataset composition was altered by incorporating observations from different seasons, enabling an out-of-season generalization assessment and preventing information leakage. In this context, the predicted values obtained through the Ridge regression model are compared with the observed values, and the model’s generalizability is analyzed. This comparative analysis based on real data provides an important reference for testing whether the model performs successfully not only on training data but also in real-world application scenarios.

The calculated performance metrics further affirm the predictive strength of the proposed model. The Proposed Method achieves the best performance on the test set (MSE = 2.92, RMSE = 1.71, MAE = 1.47), accompanied by near-perfect goodness-of-fit (R² = 0.95) and minimal normalized error (Table 7). Additionally, Scaled MSE value of 0.005 reflects that the prediction errors are quantitatively limited and relatively small in proportion to the overall data range. Overall, the evidence indicates that the Proposed Method generalizes more effectively and tracks the magnitude of the signal substantially better than the competing approaches.

**Table 7:** Additive explanations for predictions of test set cases.

Model	MSE	RMSE	MAE	*R*²	MASE
HFA–Ridge	2.920	1.710	1.470	0.950	0.383

**Fig. 7:**
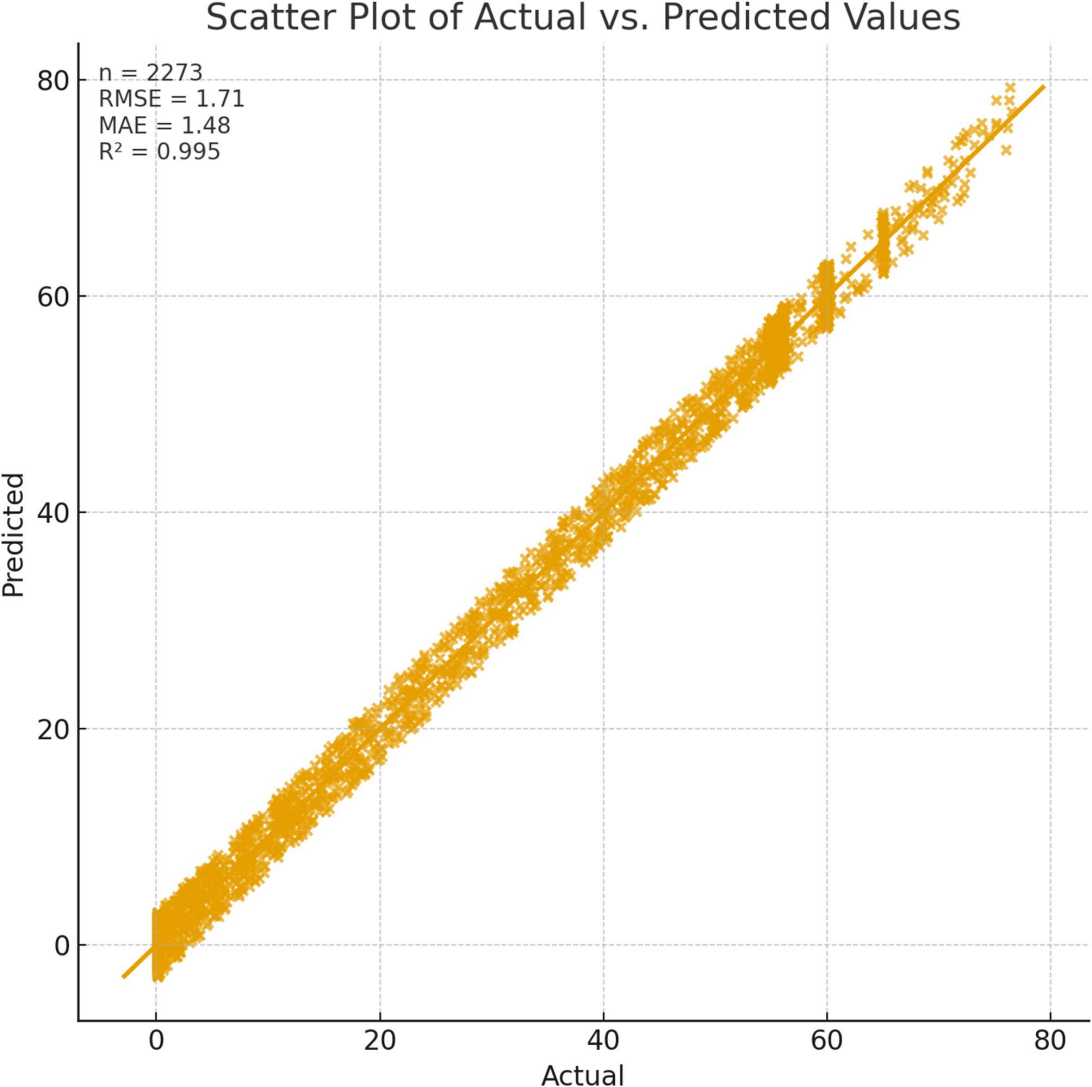
Scatter plot of actual vs. predicted values.

Figure 7 complements the quantitative performance with a visual presentation of total active power predicted and actual values in a scatter plot. Ridge Regression predictions against observed values are plotted, and a red dashed identity line depicts perfect agreement. Points lie in a tight cluster around this line throughout the full operating range, save for very few marked exceptions, which indicates slight underestimation or overestimation. This close correspondence with respect to the ideal fit shows the model gives precedence to the dominant signal, which is further supported by the low RMSE and high R² shown in Table 6, thus emphasizing that the model is robust and generalizes well in forecasting photovoltaic power from weather inputs.

**Fig. 8:**
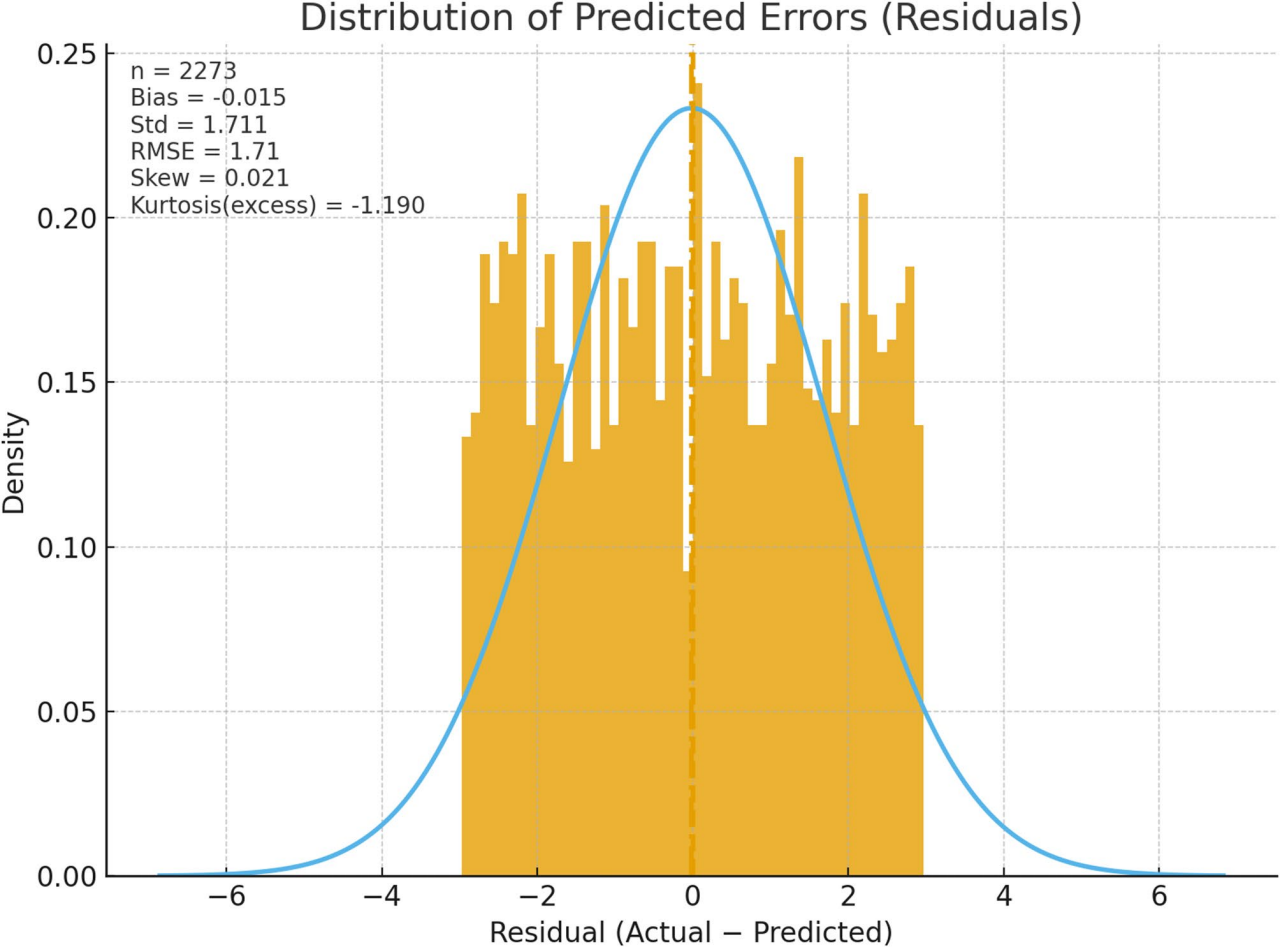
Distribution of predicted errors.

The error distribution of Fig. 8 is closely bunched around zero and appears to be nearly perfectly balanced, with no more than the odd outlier. Symmetry indicates zero systematic bias—predictions are not uniformly high or uniformly low. The near-normal shape of the histogram is in agreement with residual assumptions of a well-specified model, and the reasonably even spread suggests close to constant variance (homoskedasticity), as consistent with stable performance at both low and high power levels.

**Fig. 9:**
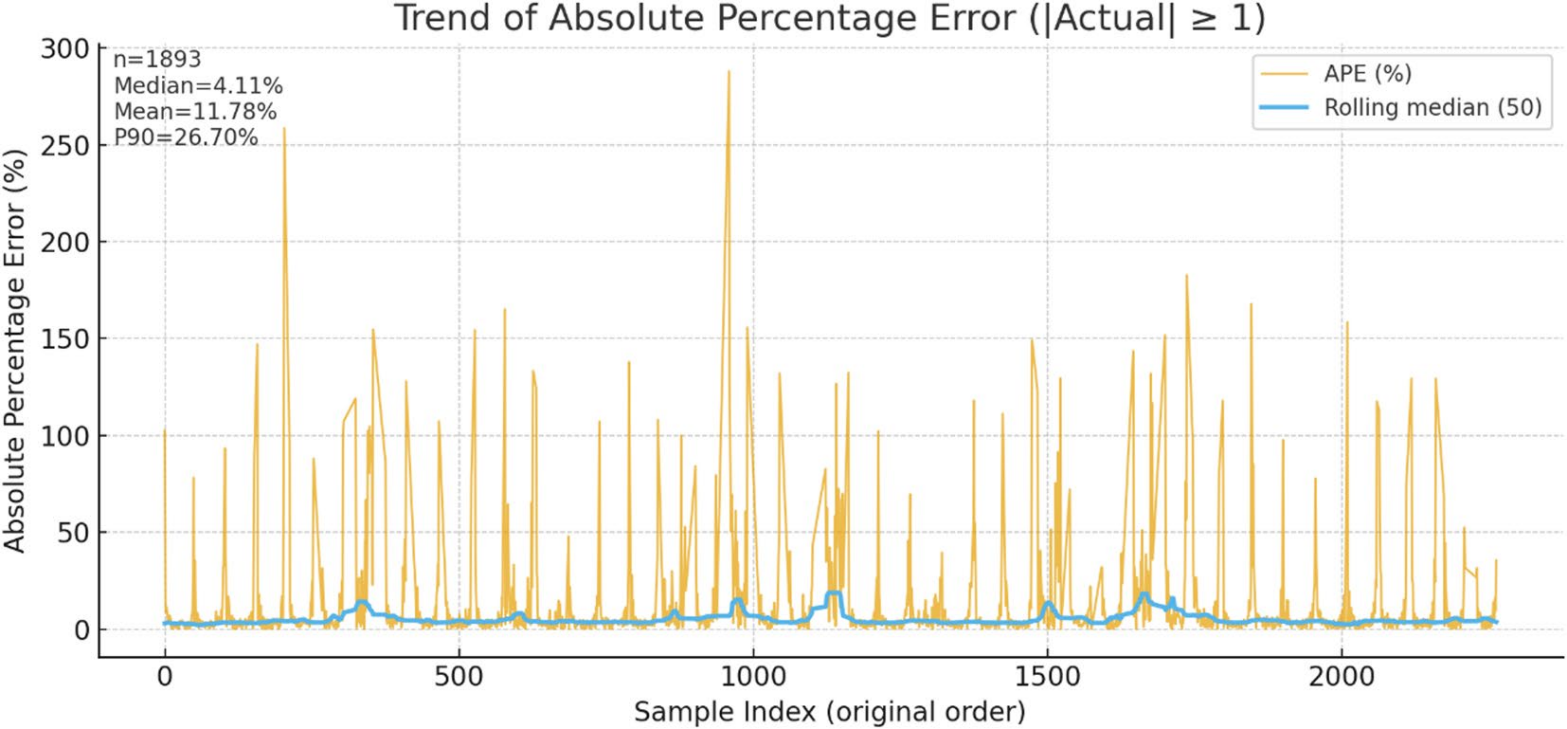
Trend of absolute percentage error.

Although the overall distribution of Absolute Percentage Error (APE) values has low and flat central tendency without visible drift, indicating consistent error behavior over the evaluation period. Regular spikes increase means that 90% of the predictions have ~ 27% relative error (Fig. 9). The sparsity and bursty nature of the peaks suggest they appear under high-ramp or low-power conditions, rather than systematic bias. In most cases, the model’s percentage errors are always low and consistent, with some high-APE cases that can be mitigated by ramp-aware features or special handling of low-power regimes.

## Benchmark comparison and result analysis

We evaluated persistence (lag‑1), ARIMA, SVR with Radial Basis Function (RBF), Random Forest, Gradient Boosting, LSTM, and the proposed HFA–Ridge under a single, time‑ordered split (train/validation/test = 0.64/0.16/0.20), with the dataset chronologically sorted by the order field and no shuffling. Pre‑processing followed train‑only fitting, standardization of covariates on train, applied to validation/test; the target was never scaled for metric computation. Hyperparameters were selected only on train + validation using blocked time‑series CV (K = 3); each model was then refit on train + validation at the selected settings and evaluated once on the held‑out test slice. We report MSE, RMSE, MAE, MASE, skill vs. persistence, and R². On the internal slice, the persistence denominators are RMSE = 6.623 kW and MAE = 3.308, which anchor all skill/MASE values (Tables 8 and 9).

**Table 8:** Tuned hyperparameters of emsemble Methods.

Model	Boosting	Random Forest	SVR
**learning_rate**	0.05	—	—
**max_depth**	2	12	—
**n_estimators**	200	200	—
**subsample**	1	—	—
**min_samples_leaf**	—	5	—
**SVR_C**	—	—	10
**SVR_epsilon**	—	—	0.2

**Table 9:** Tuned hyperparameters of LSTM.

model	window (steps)	layers	units/layer	dropout	optimizer	lr	batch	epochs (max)	early stopping
LSTM	**96**	2	64	0.1	Adam	1e-3	256	400	yes

Model-specific tuning is carried out through grid searches and parameter selection. Using the same CV protocol on train + validation:

- ARIMA orders were chosen by Akaike Information Criterion (AIC) over a bounded grid (p, q ≤ 4, d ≤ 2) with daily seasonality (s = 96) retained only if it improved AIC;

- SVR (RBF) used standardized inputs and a grid over C ∈ {1, 10, 100},zhen.

- C ∈ {1, 10, 100}, γ ∈ {“scale”, 0.1, 0.01, 0.001}, ε ∈ {0.1, 0.2, 0.3}, employing Halving Grid Search CV (factor ≈ 3) where available, otherwise Grid Search CV;

- Random Forest used n_estimators ∈ {200, 400}, max_depth ∈ {None, 12, 20}, min_samples_leaf ∈ {1, 3, 5};

- Gradient Boosting used n_estimators ∈ {200, 300, 400}, learning_rate ∈ {0.01, 0.05, 0.1}, max_depth ∈ {2, 3, 4}, subsample ∈ {0.6, 0.8, 1.0} with neg-MSE scoring;

- LSTM consumed standardized features in 96-step sliding windows (≈ 24 h − 15-min), two stacked LSTM layers (64 units each), dropout = 0.1, Adam (1e-3), batch = 256, max 400 epochs with Early Stopping/Reduce LR On Plateau;

- HFA–Ridge used three second-order latent factors (Radiation, Temperature, Wind/Climate) learned via hierarchical factor analysis, with λ chosen by CV + 1-SE rule.

Final hyperparameters actually used in Tables 10 and 8.

**Table 10:** Models performance metrics.

Model	MSE	RMSE	MAE	*R*²	MASE	skill_MSE	skill_RMSE	skill_MAE	delta_R2
ARIMA	163.251	12.570	9.208	0.761	2.784	−2.721	−0.897	−1.783	−0.152
Boosting	74.273	8.618	5.046	0.853	1.525	−0.693	−0.301	−0.525	−0.060
LSTM	255.317	15.979	10.501	0.493	3.174	−4.820	−1.412	−2.174	−0.421
Persistence	43.870	6.623	3.308	0.913	1.000	0.000	0.000	0.000	0.000
Random Forest	77.943	8.829	4.933	0.846	1.491	−0.777	−0.333	−0.491	−0.067
SVR	264.793	16.272	12.000	0.477	3.628	−5.036	−1.457	−2.628	−0.437
HFA–Ridge	104.251	10.210	7.087	0.823	2.142	−1.376	−0.541	−1.142	−0.0903

On the internal test split (Table 9), tree ensembles (Gradient Boosting, Random Forest) achieve the lowest absolute errors. Persistence is, as expected for a 15-minute horizon, a strong reference; against this anchor (RMSE = 6.623 kW; MAE = 3.308), the proposed HFA–Ridge is competitive in explained variance (R² = 0.823) yet shows negative skill in RMSE/MAE; typical in short-lag-dominated settings with high autocorrelation. ARIMA, LSTM, and SVR trail across metrics on this internal slice.

The study shows that the factor-analysis + ridge regression framework is both accurate and interpretable, offering robustness against multicollinearity and clear parameter transparency. While ensemble methods like Boosting and Random Forest achieve strong predictive performance, their complexity, overfitting risk, and low interpretability reduce their practical value. Ridge regression is highlighted as a balanced alternative, combining precision with interpretability for reliable photovoltaic forecasting.

For the validation purpose, we used a new, independent dataset distinct from the training and testing data. Specifically, the dataset was restructured by systematically selecting one full day from each month across 45 months, yielding 53 observations per day. This design ensured a homogeneous and seasonally balanced dataset, supporting fair evaluation and reducing the risk of information leakage. To test robustness under distribution shift, we froze all models and evaluated them on an independent, data set (Table 11). Ensembles show smaller gains; LSTM and SVR underperform. Taken together, these results indicate that HFA–Ridge generalizes best under seasonal shift while retaining full interpretability. It should be point out ARIMA is omitted from this Table 11 because it is a univariate baseline without exogenous inputs and does not admit feature-level additive attributions. Its predictive metrics are reported alongside the other baselines in Table 9.

**Table 11:** External multi-season validation*.

Model	MSE	RMSE	MAE	*R*²	MASE	skill_MSE	skill_RMSE	skill_MAE	ΔR²
Boosting	62.370	7.890	4.770	0.880	1.242	−0.099	−0.047	−0.242	−0.018
LSTM	106.840	10.330	6.050	0.800	1.575	−0.883	−0.371	−0.575	−0.098
Persistence	56.741	7.533	3.841	0.898	1.000	0.000	0.000	0.000	0.000
Random Forest	35.880	5.990	3.580	0.930	0.932	0.368	0.205	0.068	0.032
SVR	104.570	10.220	5.800	0.810	1.510	−0.843	−0.357	−0.510	−0.088
HFA–Ridge	2.920	1.710	1.470	0.950	0.383	0.949	0.773	0.617	0.052

*(skill vs. persistence) = 1 − (error_model/error_persistence); MASE = MAE_model/MAE_persistence;ΔR² = R²_model − R²_persistence. Positive skill and ΔR² indicate improvement over persistence; MASE < 1 likewise indicates the model outperforms persistence (MASE = 1 equals persistence).

Moreover, Table 11 illustrates how the Ridge model assigns consistent, additive contributions that align with its learned coefficients on factorized inputs. This shows clearly how physically meaningful factors translate into predictions, something black-box models cannot provide. Such clarity reinforces the value of regression-based approaches as both precise and interpretable tools for PV forecasting.

In addition, the joint evidence from Tables 9 and 11 shows a consistent trade-off: ensembles win in-split absolute error, whereas the HFA–Ridge model wins validation data set with positive skill and MASE < 1, while uniquely preserving coefficient-level interpretability and low deployment cost. Given the high multicollinearity and physical coherence among meteorological drivers, the factor-plus-ridge design yields stable coefficients, transparent case-level attributions, and favorable generalization key requirements for operational forecasting.

Finally, we analysis the computational cost and scalability of the proposed method The hierarchical factor analysis reduces the original predictor space to three second-order latent factors. Training cost is dominated by factor extraction on *p* variables, followed by a ridge fit on a 3-dimensional design. In big-O terms this is O(n·p²) for factor extraction plus O(n·k² + k³) for ridge with k = 3; inference is O(k) per time step (compute factors + one dot-product). Only a single regularization parameter λ is tuned (selected by time-series CV with the 1-SE rule), so search cost is minimal. The entire pipeline trains and serves on CPU, supports streaming deployment, and retrains rapidly for new periods or sites with the same preprocessing.

Compared with the competing baselines, the proposed HFA–Ridge is the most economical to train and redeploy: hierarchical factor analysis compresses the predictor space to three second-order factors, after which ridge is fit in a 3-D design with a single regularization parameter (λ), yielding CPU-only training and constant-time per-step inference in the reduced factor space.

- Tree ensembles (random forest, gradient boosting) require multi-parameter search over depth/trees/subsample/leaf size; their training cost grows roughly with (trees × depth × cross-validation passes), and although inference is fast, multiple tree traversals and model size complicate frequent re-training.
- SVR (RBF) incurs at least quadratic scaling in the number of samples during hyperparameter search, and prediction latency scales with the number of support vectors.

- LSTM entails epoch-based backpropagation over sequences with a larger hyperparameter surface (layers/units/dropout/batch/optimizer) and typically benefits from GPU acceleration; inference is linear in sequence length yet heavier than a single linear model.

- Classical ARIMA needs order/seasonality selection and becomes costly under repeated re-fitting or seasonal terms. As a result, while tree ensembles attain the lowest in-split absolute errors,

- HFA–Ridge offers the most favorable accuracy–interpretability–cost trade-off—minimal tuning (one λ), compact CPU-only deployment, and positive external performance (MASE < 1, positive skill) on the independent multi-season set—hence it scales best for operational use. Scalability is analyzed analytically via algorithmic complexity and tuning burden and linked empirically to external performance.

## Discussion and conclusion

This study introduces an interpretable, statistically grounded two-stage forecasting framework that combines HFA with ridge regression. By organizing meteorological drivers into a small set of second-order latent factors and regularizing the final linear model, the approach addresses multicollinearity explicitly while preserving coefficient-level interpretability. The resulting representation is conceptually coherent and computationally lean, offering a structured alternative to flat, high-dimensional inputs commonly used in PV forecasting.

The benchmarking results, read jointly across the internal split (Table 8) and the independent data set (Table 9), reveal a consistent pattern. On the internal 15-minute horizon, the lag-1 persistence baseline is strong and tree ensembles achieve the lowest absolute errors; the proposed HFA–Ridge attains competitive explained variance (R² ≈ 0.82) but shows negative skill vs. persistence in RMSE/MAE—an outcome consistent with short-lag-dominated settings. Crucially, when evaluated out-of-season without refitting, HFA–Ridge outperforms all baselines on the external set, achieving MASE < 1 and positive skill vs. persistence (Table 9). This indicates superior transportability to unseen seasonal regimes, which is often the decisive criterion for operational deployment.

From a practical standpoint, the method is inexpensive to train, simple to redeploy, and auditable. HFA compresses correlated inputs to three physically meaningful factors and ridge regression tunes only a single regularization parameter; the entire pipeline runs on CPU with constant-time per-step inference in the reduced factor space. In contexts where retraining frequency, resource constraints, or regulatory accountability matter the combination of external-set accuracy, parameter transparency, and low operational cost provides a favorable accuracy–interpretability–deployment trade-off. While ensembles remain attractive for minimizing in-split error, their tuning burden, model size, and limited coefficient-level interpretability can be constraining in field settings; conversely, HFA–Ridge is slightly behind in-split yet best under seasonal shift and far easier to scale across sites and periods.

In sum, the proposed HFA-Ridge design provides an accurate, generalizable, and explainable forecasting pipeline that aligns scientific interpretability with operational needs. It complements high-performing ensembles by offering a transparent, low-cost option that excels under seasonal shift, thereby broadening the toolkit available for reliable PV power forecasting.

## Data Availability

The datasets generated and/or analysed during the current study are available in the GitHub repository: https://github.com/tkaraca/An-Interpretable-Statistical-Approach-to-Photovoltaic-Power-Forecastin.git.

## Abbreviations

ANN: Artificial Neural Networks
AIC: Akaike Information Criterion
BIPV: Building Integrated Photovoltaic
DL: Deep Learning
ELM: Extreme Learning Machines
FSPV: Floating Solar Photovoltaic
GPR: Gaussian Process Regression
GRU: Gated Recurrent Units
HFA: Hierarchical Factor Analysis
LSTM: Long Short-Term Memory
ML: Machine Learning
PCA: Principal Component Analysis
PV: Photovoltaic
RBF: Radial Basis Function
RE: Renewable Energy
RNN: Recurrent Neural Networks
SVM: Support Vector Machines
TSFA: Time Series Factor Analysis
